# Emerging CSF and Serum Biomarkers in Multiple Sclerosis: Cytokines, MOG, GFAP, and Beyond

**DOI:** 10.1212/NXI.0000000000200578

**Published:** 2026-04-23

**Authors:** Johanna Oechtering, Sabine Anna Schaedelin, Aleksandra Maleska Maceski, Kerstin Stein, Nafiye Genc, Maximilian Einsiedler, Pascal Benkert, Eline Willemse, Tobias J. Derfuss, Bettina Fischer-Barnicol, Marcus D'Souza, Axel Regeniter, Wayne Hu, Ferhan Qureshi, Sebastian Finkener, Stefanie Mueller, Robert Hoepner, Patrice H. Lalive, Caroline Pot, Claudio Gobbi, Chiara Zecca, Patrick Roth, Heinz Wiendl, Jan D. Lünemann, Ludwig Kappos, Cristina Granziera, David Leppert, Jens Kuhle, as the Swiss MS Cohort Study

**Affiliations:** 1 Department of Neurology, University Hospital and University of Basel, Switzerland;; 2 Multiple Sclerosis Centre and Research Center for Clinical Neuroimmunology and Neuroscience (RC2NB), Departments of Biomedicine and Clinical Research, University Hospital and University of Basel, Switzerland;; 3 Department of Neurology and Neurophysiology, Medical Center, University of Freiburg, Faculty of Medicine, University of Freiburg, Germany;; 4 Clinical Trial Unit, Department of Clinical Research, University Hospital Basel, University of Basel, Switzerland;; 5 Department of Neurology, University Hospital Münster, Germany;; 6 Medica Laboratory, Zürich, Switzerland;; 7 Octave Bioscience, Menlo Park, CA;; 8 Department of Neurology, Cantonal Hospital, Aarau, Switzerland;; 9 Department of Neurology, Cantonal Hospital St. Gallen, University Teaching and Research Hospital, HOCH Health Ostschweiz, Switzerland;; 10 Department of Neurology, Inselspital, Bern University Hospital and University of Bern, Switzerland;; 11 Department of Clinical Neurosciences, Division of Neurology, Geneva University Hospital, Switzerland;; 12 Diagnostic Department, Division of Laboratory Medicine, Geneva University Hospital, Switzerland;; 13 Department of Pathology and Immunology, Faculty of Medicine, University of Geneva, Switzerland;; 14 Division of Neurology, Department of Clinical Neurosciences, Lausanne University Hospital (CHUV) and University of Lausanne, Switzerland;; 15 Neurocentre of Southern Switzerland, Multiple Sclerosis Centre, Ospedale Civico, EOC, Lugano, Switzerland;; 16 Faculty of Biomedical Sciences, Università della Svizzera Italiana (USI), Lugano, Switzerland;; 17 Department of Neurology, University Hospital Zurich and University of Zurich, Switzerland; and; 18 Translational Imaging in Neurology (ThINk) Basel, Department of Biomedical Engineering, Faculty of Medicine, University Hospital Basel and University of Basel, Switzerland.

## Abstract

**Background and Objectives:**

Accurate biomarkers that reflect disease activity, severity, and molecular pathophysiology in multiple sclerosis (MS) remain an unmet diagnostic need. We compared the performance of the established biomarkers, neurofilament light chain (NfL) and glial fibrillary acidic protein (GFAP), with emerging candidates in CSF and serum.

**Methods:**

We measured 21 analytes using Olink proximity extension assay technology, NfL and GFAP using Simoa, and soluble triggering receptor expressed on myeloid cells (sTREM2) and neuronal pentraxin 2 (NPTX2) using Fujirebio platforms in paired CSF and serum/plasma samples from 293 participants. The cohort included patients with clinically isolated syndrome (CIS), relapsing-remitting MS (RRMS), primary progressive MS (PPMS), and secondary progressive MS (SPMS), as well as individuals with inflammatory neurologic disease and symptomatic controls (SCs). CSF and serum biomarker levels were compared across diagnostic groups using multivariable Cox and linear regression models. Associations were assessed using CSF immunoglobulin profiles, time from first to second clinical event, and Expanded Disability Status Scale (EDSS) scores in CIS, as well as the Multiple Sclerosis Severity Score (MSSS) in patients with MS.

**Results:**

Eight biomarkers showed consistent differential expression. In CSF, CXCL13, CXCL9, IL-12b, and NfL were elevated in most CIS and MS subgroups compared with SCs. Osteopontin (OPN) levels were increased in RRMS and PPMS subgroups, whereas TNFRSF10A elevations were confined to patients with PPMS. In serum, NfL was elevated across all CIS and MS subgroups, GFAP was increased in RRMS and SPMS subgroups, and myelin oligodendrocyte glycoprotein (MOG) was increased in RRMS and PPMS subgroups. Higher CSF levels of CXCL13, CXCL9, and IL-12b predicted shorter intervals to a second clinical attack and correlated strongly with intrathecal IgM synthesis. Elevated EDSS scores at CIS onset were linked to higher CSF levels of CXCL13, IL-12b, TNFRSF10A, and OPN, and to NfL, GFAP, and MOG in serum. Prediction of future MSSS was limited to GFAP in CSF, whereas in serum, GFAP, MOG, OPN, and CXCL9 were significantly associated.

**Discussion:**

CSF CXCL13, CXCL9, and IL-12b are promising biomarkers for predicting relapse activity, while serum GFAP and MOG appear to be consistent candidates for prognosticating disease severity.

## Introduction

Multiple sclerosis (MS) is a chronic, autoimmune-mediated disease of the CNS that leads to neuronal cell loss, demyelination, and astrocyte proliferation; its clinical presentation and long-term outcome are highly variable across patients.^1^ Because the actual clinical symptomatology is an unreliable measure to monitor concurrent inflammatory activity and to anticipate future disease severity, biomarkers are urgently needed to support therapeutic decision making.^2^ Neurofilament light chain (NfL) and glial fibrillary acidic protein (GFAP) are currently the best validated biomarkers for this purpose: NfL primarily reflects neuroaxonal damage due to focal inflammation and correlates clinically with relapse-associated worsening, while GFAP is associated with astrocyte activation and astrogliosis in the course of scar formation leading to disease progression independent of relapse activity (PIRA).^3-5^

Using proximity extension assay (PEA) technology, 21 of >1,400 serum proteins had recently been identified to be associated with increased clinical and radiographic MS disease activity for inclusion in a custom assay panel (Octave).^6,7^ These biomarkers reflect different aspects of MS pathophysiology: cytokines such as CXCL13, CXCL9, IL-12b, CD6, CCL20, BAFF (TNFSF13B), and osteopontin (OPN) are mainly involved in regulating various cellular immune responses. The TNF receptor TNFRSF10A mediates apoptosis, while myelin oligodendrocyte glycoprotein (MOG), a transmembrane protein expressed exclusively on oligodendrocytes and the outer CNS myelin sheath, serves as a marker of CNS myelin damage. Other candidates were NfL, GFAP, VCAN, COL4A1, FLRT2, OPG, SERPINA9, GH, PRTG, APLP1, CNTN2, and CDCP1 (detailed description in eTable 1). We added soluble triggering receptor expressed on myeloid cells 2 (sTREM2) and neuronal pentraxin 2 (NPTX2) as 2 other potential biomarkers reflecting microglial activation and mediating neuronal plasticity, respectively, to the analysis panel.

We aimed to investigate the capacity of these biomarkers to assess MS disease activity and to anticipate future disability accumulation in serum and CSF. We analyzed the correlation of biomarker levels between the 2 biofluid compartments, comparing across MS subgroups and disease stages, with symptomatic controls (SCs) and inflammatory neurologic disease controls (INDCs) serving as references.^8^ Furthermore, we analyzed their relationship with measures of intrathecal humoral immune response and evaluated their predictive value for future relapses and associations with the Expanded Disability Status Scale (EDSS) in CIS and the future Multiple Sclerosis Severity Score (MSSS).^9^

## Material and Methods

### Patients, Inclusion Criteria, and Data Collection

CSF and serum/plasma samples were obtained from the CSF biobank of the University Hospital Basel, and a subset of patients were prospectively recruited into the Swiss multiple sclerosis cohort (SMSC) between 2012 and 2024.^10^ Patients included had clinically isolated syndrome (CIS),^11^ relapsing-remitting multiple sclerosis (RRMS),^11^ secondary progressive multiple sclerosis (SPMS),^12^ or primary progressive multiple sclerosis (PPMS).^13^ Because diagnostic classifications evolved over the study period, and to distinguish between clinically definite multiple sclerosis (defined by at least 2 clinical relapses) and very early disease stages, patients were categorized as RRMS after the occurrence of 2 relapses, whereas patients after a first demyelinating event were called “CIS” for simplicity according to historical classifications.

We further included inflammatory neurologic disease controls (INDCs) as well as symptomatic controls (SCs)^8^ without structural CNS disease. CSF samples were centrifuged at 400×*g* for 10 minutes within 30 minutes of collection. Serum samples were collected concurrently, centrifuged at 2,000×*g* for 10 minutes, and the supernatants were immediately frozen at −80°C. A maximum of one thaw-freeze cycle was permitted in a subset of samples for realiquotation purposes. Neurologic symptoms that lasted for ≥24 hours without fever, infection, or adverse reaction to a prescribed medication were defined as first symptoms.^13^ Patients classified as RRMS had to have experienced at least 2 relapses before the lumbar puncture (LP).^13^ Demographic and clinical variables collected included sex, date of birth and CIS/MS onset of first symptoms, disease-modifying treatment history, and EDSS score at LP and at last follow-up visit. In controls, the specific diagnoses were collected.

### Biomarker Measurements

We measured CSF and serum GFAP/NfL levels in duplicate using the Neurology 2-PLEX B assay (Quanterix, Billerica, MA, USA). The mean intra-assay coefficients of variation (CVs) of duplicate determinations for GFAP and NfL were 2.3 and 2.5%. The interassay CVs of internal QCs for GFAP were 8.0% (73.6 pg/mL), 6.7% (127.3 pg/mL), and 6.9% (325.9 pg/mL) and for NfL were 6.8% (8.7 pg/mL), 4.4% (17.9 pg/mL), and 6.1% (106.5 pg/mL). CSF and serum samples were assessed using the Octave Multiple Sclerosis Disease Activity (MSDA) Test custom assay panel, a multiprotein biomarker assay using Olink^®^ PEA methodology, including 21 analytes, 18 of which were selected for incorporation into the MSDA Test algorithm (eTable 1).^6,7^ Mean intra-assay CVs of duplicate determinations and the mean interassay CVs for all biomarkers are listed in eTables 2 and 3. sTREM2 and NPTX2 were determined in CSF and plasma using Fujirebio Innotest assays (catalogue number #81056 for sTREM2 and #80908 for NPTX2) according to the manufacturer's recommendations.

For data points below the lower limit of quantification (LLOQ), a random value between zero and LLOQ was imputed. For data points above the upper limit of quantification (ULOQ), we used the ULOQ (CSF (c)CXCL9: 0.9%; cGFAPOlink: 0.9%). In addition, for cNfL, Olink we used a higher dilution (1:100) than what had previously been validated for serum. The numbers per biomarker are listed in eTable 4.

### Statistics

Patients and clinical data are presented as median and interquartile range (IQR) for continuous variables and as absolute and relative frequencies in case of categorical data. The distribution of the biomarkers was inspected using QQ plots. The fluid biomarker levels were log-transformed prior to analysis, to better comply with normal assumption. To facilitate the estimate interpretation, a logarithm with base 2 was used in those analyses including the biomarkers as independent variables.

Differences in CSF or serum/plasma biomarker levels between CIS, RRMS, SPMS, PPMS, and INDC vs SC subgroups (independent variables) were analyzed using linear regression models. A separate model was fit for each biomarker. Each model was adjusted for age, sex, and albumin quotient (Qalb).

Spearman rank correlations (r) were calculated to quantify the CSF-serum/-plasma correlations for individual biomarkers as well as to assess correlations between NfL and GFAP measurements obtained by 2 different platforms (Simoa and Octave MSDA Test) across all samples (n = 293). In addition, Spearman r correlations were evaluated among patients with CIS and MS (n = 233) for all combinations of biomarkers in both CSF and serum/plasma. Furthermore, principal component analysis was performed to summarize shared variance among biomarkers to assess patterns of collinearity in a multivariable manner. We analyzed associations of the CSF immunoglobulin (Ig) categories with biomarker levels in CIS and MS combined. Because intrathecal synthesis of Ig subtypes in MS is not evenly and independently distributed, and intrathecal IgG and IgM synthesis, as defined by the Reiber formula, does not occur independently of oligoclonal IgG bands, we applied a stepwise categorization to capture the extent and layering of the humoral immune response. Patients were categorized based on the presence or absence of oligoclonal IgG bands (OCGBs), IgG_IntrathecalFraction(IF)_, and IgM_IF_, using thresholds of >0% vs 0%, as described previously.^14,15^ The following groups were defined:

1. OCGB^−^/IgG_IF_^−^/IgM_IF_^−^; n = 42,
2. OCGB^+^/IgG_IF_^−^/IgM_IF_^−^; n = 50,
3. OCGB^+^/IgG_IF_^+^/IgM_IF_^−^; n = 77, and
4. OCGB^+^/IgG_IF_^+^/IgM_IF_^+^; n = 55

Five patients (1.7%) were IgG_IF_^+^/or IgM_IF_^+^ in combination with OCGB^−^, and 6 patients (2.0%) had an OCGB^+^/IgG_IF_^−^/IgM_IF_^+^ profile and were excluded from this analysis. Using category 1 as reference, associations of the CSF Ig categories (2) to (4) (independent variables) with the 23 analytes (dependent variable, in CSF or serum, respectively) were investigated using linear regression models adjusted for age, sex, Qalb, and DMT category at LP (platform, orals, monoclonal antibodies vs untreated, respectively; comparison of the categorization in eTable 5).

Time to second clinical event and EDSS score at first demyelinating event were evaluated in patients with CIS only using Cox proportional hazards models and linear models, respectively. Each biomarker was used as an independent variable in a separate model. The linear models were adjusted for age, sex, and Qalb while DMT category was used as additional time-varying covariate in the Cox proportional hazards models.

Future MSSS, based on EDSS scores at the last follow-up visit, was analyzed in CIS and MS combined using a linear model. Again, the biomarker served as independent variable, and a separate model was fit for each biomarker. The model was adjusted for age, sex, and Qalb, as well as the dominant treatment category, the category the patient was treated for the most time, was included as the adjusting variable.

Estimates were back-transformed to indicate the effect in percentage and hazard ratios in case of the Cox proportional models. It should be noted that the focus of these analyses was on patterns of association across related biomarkers and the consistency of findings, rather than on individual *p* values or binary significance thresholds, reflecting our interest in biologically meaningful trends rather than formal hypothesis testing. Statistical analyses were conducted using R (version 3.6.3).

### Standard Protocol Approvals, Registrations, and Patient Consents

A subset of patients from the CSF biobank study of the Department of Neurology, University Hospital Basel, were prospectively recruited into the SMSC. Studies were approved by the local ethical committee after written informed consent.

### Data Availability

Data are available on reasonable request.

## Results

### Patients and Clinical Data

CSF and blood samples from 233 patients (110 with CIS, 87 with RRMS, 23 with SPMS, 13 with PPMS) and 60 controls (30 SCs, 30 INDCs) (eTable 6) were included. Patients with SPMS were significantly older and had a longer disease duration and higher EDSS scores at LP; most patients with CIS/MS (87.6%) were untreated at LP (Table 1).

**Table 1:** Demographic, Clinical, and CSF Characteristics at Lumbar Puncture

	CIS	RRMS	SPMS	PPMS	SC	INDC
n	110	87	23	13	30	30
Demographic and clinical data						
Female (n, %)	78 (70.9)	63 (72.4)	13 (56.5)	7 (53.8)	21 (70.0)	14 (46.7)
Age at LP (y)	34.4 (26.7–43.5)	36.9 (30.8–48.0)	53.7 (48.9–59.6)	48.7 (48.1–58.8)	35.5 (21.9–49.9)	54.3 (36.0–71.5)
Disease duration at LP (m)	0.6 (0.3–1.9)	55.0 (15.3–90.7)	253.1 (147.0–332.1)	43.6 (12.6–66.8)	—	—
EDSS score at LP	2.0 (1.5–2.5)	2.5 (2.0–3.5)	6.0 (4.0–7.0)	3.5 (3.0–4.0)	—	—
Untreated (n, %)	109 (99.1)	68 (78.2)	15 (65.2)	12 (92.3)	—	—
Follow-up available (n, %)	87 (79.1)	62 (71.3)	19 (82.6)	8 (61.5)	—	—
Follow-up time (y)	6.4 (4.0–9.1)	6.4 (3.8–9.6)	6.0 (4.3–7.1)	7.4 (5.1–9.3)	—	—
McDonald Criteria 2017, yes (%)	70 (63.6)	87 (100)	23 (100)	13 (100)	—	—
CSF data						
Cell count	4.4 (2.0–9.0)	3.0 (1.0–6.0)	1.0 (0.3–2.2)	1.7 (1.0–3.0)	1.0 (0.7–2.0)	25.0 (6.5–76.5)
Q_alb_	4.5 (3.6–6.4)	5.3 (3.9–7.4)	5.5 (3.8–7.7)	5.1 (4.4, 7.3)	4.2 (3.7–5.0)	9.6 (7.7, 12.4)
OCGB^+^ (%)	88 (80.0)	71 (82.6)	17 (85.0)	11 (84.6)	0 (0)	11 (37.9)
IgG_IF_^+^ (%)	64 (58.2)	51 (59.3)	9 (45.0)	8 (61.5)	0 (0)	7 (24.1)
IgM_IF_^+^ (%)	31 (28.2)	24 (27.9)	2 (10.0)	3 (23.1)	0 (0)	7 (24.1)
IgA_IF_^+^ (%)	5 (4.5)	7 (8.1)	1 (5.0)	1 (7.7)	0 (0)	3 (10.3)
OCGB^−^/IgG_IF_^−^/IgM_IF_^−^ (%)^a^	22 (20.6)	15 (17.9)	3 (13.0)	2 (15.4)	30 (100.0)	17 (60.7)
OCGB^+^/IgG_IF_^−^/IgM_IF_^−^ (%)^a^	21 (19.6)	18 (21.4)	8 (34.8)	3 (23.1)	0 (0)	4 (14.3)
OCGB^+^/IgG_IF_^+^/IgM_IF_^−^ (%)^a^	36 (33.6)	29 (34.5)	7 (30.4)	5 (38.5)	0 (0)	1 (3.6)
OCGB^+^/IgG_IF_^+^/IgM_IF_^+^ (%)^a^	28 (26.2)	22 (26.2)	2 (8.7)	3 (23.1)	0 (0)	6 (21.4)

Abbreviations: CIS = clinically isolated syndrome; Ig G/M/A_IF_ = immunoglobulin G/M/A intrathecal fraction; INDC = inflammatory neurologic disease control; IQR = interquartile range; m = month; OCGB = oligoclonal IgG band; PPMS = primary progressive MS; Q_alb_ = albumin quotient; RRMS = relapsing-remitting MS; SC = symptomatic control; SPMS = secondary progressive MS, y = years.

Median and IQR are displayed if not mentioned otherwise.

^+^: Presence of OCGB or IgG_IF_/IgM_IF_/IgA_IF_.

^−^: Absence of OCGB or IgG_IF_/IgM_IF_/IgA_IF_.

a 5 (1.7%) OCGB^−^/IgG_IF_^+^ or OCGB^−^/IgM_IF_^+^ patients (1 with RRMS, 3 with SPMS, 1 INDC) and 6 (2.0%) OCGB^+^/IgG_IF_^−^/IgM_IF_^+^ patients (3 with CIS, 2 with RRMS, 1 INDC) were excluded because they did not fit into classifications.

### Group Differences Between CIS/MS Subgroup and INDCs and SCs

#### CSF

##### Cell Structure Proteins

**Figure 1:**
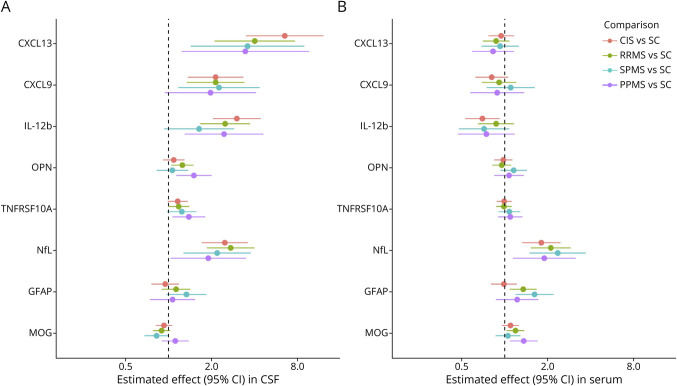
Multivariable Analysis Comparing Levels of Selected Biomarkers in CIS, RRMS, SPMS, and PPMS Groups vs Symptomatic Controls (A) CSF: CXCL13, CXCL9, and IL-12b were elevated in almost all MS subgroups, prominently in CIS (2.1- to 6.5-fold, all *p* < 0.001). OPN was increased 1.2-fold (*p* = 0.016) in RRMS and 1.5-fold (*p* = 0.005) in PPMS. TNFRSF10A was elevated 1.4-fold (*p* = 0.005) in the PPMS group, and NfL was similarly increased in all MS subgroups. GFAP and MOG were not elevated in MS. (B) Serum: CXCL13, CXCL9, IL-12b, OPN, and TNFRSF10A were not elevated. NfL was elevated in all MS subgroups; GFAP was increased 1.4-fold in patients with RRMS (*p* < 0.01), 1.6-fold in patients with SPMS (*p* < 0.01), and MOG 1.2-fold in patients with RRMS (*p* = 0.016), and 1.4-fold in patients with PPMS (*p* = 0.007). CIS = clinically isolated syndrome; GFAP = glial fibrillary acidic protein; MOG = myelin oligodendrocyte glycoprotein; NfL = neurofilament light chain; OPN = Osteopontin; PPMS = primary progressive MS; RRMS = relapsing-remitting MS; SC = symptomatic control; SPMS = secondary progressive MS.

NfL levels were consistently elevated in all 4 MS subgroups and in INDCs vs SCs. GFAP levels were 1.4-fold higher in INDCs (*p* = 0.025) compared with SCs, but there were no significant elevations between the MS subgroups; the same was the case for MOG (Figure 1A; eTable 7).

##### Chemokines and Cytokines

Levels of CXCL13, CXCL9, and IL-12b were elevated across all MS subgroups vs SCs, except for CXCL9 in patients with PPMS and IL-12b in patients with SPMS where there was only a trend. The increase was most pronounced in patients with CIS vs SCs (2.1–6.5-fold higher, *p*_all_ < 0.001); levels of CXCL13 and IL-12b, but not of CXL9, exceeded those of the other MS subgroups. In the INDC group, CXCL13, CXCL9, and IL-12b levels were elevated 15.3, 26.1, and 7.5-fold, respectively (*p*_all_ < 0.0001). OPN concentrations were increased by 1.2-fold (*p* = 0.016) in patients with RRMS and 1.5-fold (*p* = 0.005) in patients with PPMS. TNFRSF10A levels were elevated by a factor of 1.4 (*p* = 0.005) in patients with PPMS and 1.7 (*p* < 0.0001) in INDCs and showed a consistent trend in the other MS subgroups (*p* ≤ 0.075) (Figure 1A; eTable 7).

##### Adhesion Molecules and Other Compounds

CNTN2 levels and VCAN concentrations were both decreased in patients with SPMS (0.8-fold, *p* = 0.020, and 0.7-fold, *p* = 0.013) and in INDCs (0.8-fold, *p* = 0.037, and 0.8-fold, *p* = 0.021). No significant differences were observed for COL4A1, OPG, APLP1, FLRT2, CDCP1, PRTG, sTREM2, or NPTX2 among the different diagnostic groups (eTable 7).

CSF TNFSF13B, SERPINA9, GH, CCL20, and CD6 levels were excluded from the analyses because 37.9%, 49.5%, 42.3%, 82.6%, and 78.5% of measurements fell below the lower limit of quantification (eTable 4).

#### Serum

##### Cell Structure Proteins

Serum (s)NfL levels were elevated in all MS subgroups as well as in INDCs. In INDCs, sGFAP concentrations were 1.9-fold higher vs SCs (*p* < 0.001). Different from CSF, levels of sGFAP levels were increased in patients with RRMS (1.4-fold, *p* < 0.01) and in patients with SPMS (1.6-fold, *p* < 0.01), as well as those of MOG in patients with RRMS (1.2-fold; *p* = 0.016), those with PPMS (1.4-fold; *p* = 0.007), and INDCs (1.3-fold; *p* = 0.007) (Figure 1B; eTable 8).

##### Chemokines, Cytokines, Adhesion Molecules, and Other Compounds

In contrast to CSF, serum levels of CXCL13, CXCL9, IL-12b, OPN, and TNFRSF10A were not increased in any MS subgroup, whereas in INDCs, CXCL9 levels were 2.1-fold (*p* < 0.001), OPN 1.4-fold (*p* = 0.0005), and TNFRSF10A 1.4-fold higher (*p* = 0.0004) vs SCs (Figure 1B; eTable 8). VCAN concentrations were 1.2-fold higher in both patients with SPMS (*p* = 0.003) and INDCs (*p* = 0.001). SERPINA9 levels were decreased in the CIS group (0.7-fold; *p* = 0.019). COL4A1 and OPG were 1.3-fold (*p* = 0.031) and 1.2-fold (*p* = 0.014) higher in INDCs, but no differences were found across the MS subgroups. No significant differences in levels were observed among the different diagnostic groups for TNFSF13B, CNTN2, OPG, APLP1, CCL20, CD6, FLRT2, CDCP1, GH, or PRTG, sTREM2, and NPTX2 (eTable 8).

### Correlations Between Biomarkers

#### Correlations of Individual Biomarkers Between CSF and Serum/Plasma

The strongest CSF-serum (plasma) Spearman *r* correlations were observed for NfL (*r* = 0.69; *p* < 0.001), followed by GFAP (*r* = 0.52; *p* < 0.001). Higher CSF-serum (plasma) correlations (*r* > 0.20) were also found for CXCL9 (r = 0.32),TNFRSF10A (r = 0.32), PRTG (*r* = 0.32), COL4A1 (*r* = 0.27), FLRT2 (r = 0.27), CNTN2 (*r* = 0.26), IL-12b (r = 0.25), OPG (*r* = 0.22), and CDCP1 (*r* = 0.21) (*p*_all_ < 0.001), while MOG (*r* = 0.04; *p* = 0.502) and OPN (*r* = 0.10; *p* = 0.134) levels were not correlated between these 2 fluid compartments (numeric data provided in eTable 9; eFigure 1).

For technical validation, we compared measurements of NfL and GFAP between Simoa and Octave MSDA Test: NfL measurements showed an almost perfect correlation between the 2 assay platforms, in both CSF (*r* = 0.95; *p* < 0.0001) and serum (*r* = 0.95; *p* < 0.0001); the same was the case for GFAP (CSF: *r* = 0.84 and serum: *r* = 0.88; both *p* < 0.0001).

#### Correlations Between Biomarkers and Principal Component Analysis in CSF

CSF levels of CXCL13, CXCL9 with IL-12b, and OPN as well as TNFRSF10A with NfL were consistently intercorrelated with each other. Moreover, CXCL9 was correlated with GFAP and sTREM2 (full correlation matrix in eFigure 2). Largely consistent with the bivariate correlation results, the PCA demonstrated substantial shared variance among the strongest biomarkers (CXCL13, CXCL9, and IL-12b) (eFigure 3).

#### Correlations Between Biomarkers and Principal Component Analysis in Serum

NfL, GFAP, and MOG serum levels were prominently intercorrelated, and especially, MOG and GFAP featured similar correlation patterns with other biomarkers such as OPN, VCAN, TNFSFR10A, and sTREM2 (eFigure 4). Also largely in line with bivariate correlation results, the PCA demonstrated substantial shared variance among NfL, GFAP, MOG, OPN, VCAN, and APLP1 (eFigure 5).

### Associations of Biomarkers With CSF Immunoglobulin Categories

#### CSF

**Figure 2:**
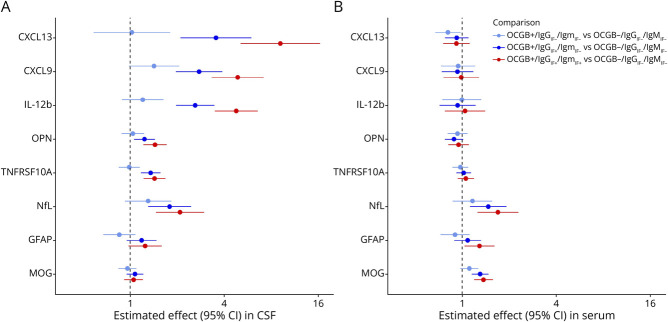
Associations of Biomarker Candidates in CSF (A) and Serum (B) With CSF Immunoglobulin Categories by Multivariable Analyses in CIS/MS (A) In CSF, especially CXCL13, CXCL9, and IL-12b showed a strong, stepwise increase in association with the spectrum of intrathecal IgG synthesis, with the most significant increases occurring when intrathecal IgM synthesis was also present. A similar, but less pronounced, pattern was observed for CSF concentrations of OPN, TNFRSF10A, and NfL. (B) Like the pattern in CSF, serum levels of NfL, GFAP, and MOG were increased in the presence of an intrathecal IgG synthesis and were maximal with additional IgM^+^. CIS = clinically isolated syndrome; GFAP = glial fibrillary acidic protein; Ig G/M/A_IF_ = immunoglobulin G/M/A intrathecal fraction; MOG = myelin oligodendrocyte glycoprotein; NfL = neurofilament light chain; OCGB = oligoclonal IgG band; OPN = osteopontin; ^+^: presence of OCGB or IgG_IF_/IgM_IF_/IgA_IF_; ^-^: absence of OCGB or IgG_IF_/IgM_IF_/IgA_IF_.

Levels of CXCL13, CXCL9, and IL-12b showed a strong, stepwise increase in association with the spectrum of intrathecal IgG synthesis, with the most significant increases occurring when intrathecal IgM synthesis was present. For instance, CXCL13 levels in CSF were 3.5-fold higher in the OCGB^+^/IgG^+^/IgM^−^ category and 9.2-fold higher (both *p* < 0.0001) in the OCGB^+^/IgG^+^/IgM^+^ category, compared with patients with MS without the presence of CSF-specific oligoclonal IgG bands (OCGB^−^/IgG^−^/IgM^−^). A similar pattern, albeit with smaller increases, was observed for the CSF concentrations of OPN, TNFRSF10A, FLRT2, and NfL, the latter aligning with previously published data^14,15^(Table 2; Figure 2A). Conversely, CSF levels of GFAP and MOG were not increased in function of types of intrathecal Ig production.

**Table 2:** Multivariable Associations* of CSF and Serum Biomarkers With CSF Immunoglobulin Groups

	**	CSF n = 224			Serum n = 224		
		Est.	CI	*p* Value	Est.	CI	*p* Value
CXCL13	OCGB^+^/IgG^−^/IgM^−^	1.02	0.58; 1.80	0.9328	0.81	0.67; 0.98	**0.0293**
	OCGB^+^/IgG^+^/IgM^−^	3.54	2.10; 5.98	**< 1e-04**	0.92	0.78; 1.10	0.3640
	OCGB^+^/IgG^+^/IgM^+^	9.16	5.10; 16.46	**< 1e-04**	0.92	0.76; 1.12	0.3914
CXCL9	OCGB^+^/IgG^−^/IgM^−^	1.42	0.98; 2.06	0.0636	0.94	0.73; 1.21	0.6417
	OCGB^+^/IgG^+^/IgM^−^	2.76	1.96; 3.90	**< 1e-04**	0.93	0.74; 1.18	0.5603
	OCGB^+^/IgG^+^/IgM^+^	4.88	3.33; 7.17	**< 1e-04**	0.99	0.76; 1.28	0.9170
IL-12b	OCGB^+^/IgG^−^/IgM^−^	1.20	0.89; 1.63	0.2355	1.00	0.75; 1.33	0.9740
	OCGB^+^/IgG^+^/IgM^−^	2.61	1.97; 3.46	**< 1e-04**	0.93	0.72; 1.22	0.6190
	OCGB^+^/IgG^+^/IgM^+^	4.78	3.48; 6.57	**< 1e-04**	1.04	0.77; 1.40	0.7850
OPN	OCGB^+^/IgG^−^/IgM^−^	1.04	0.88; 1.23	0.6416	0.93	0.81; 1.08	0.3560
	OCGB^+^/IgG^+^/IgM^−^	1.24	1.06; 1.44	**0.0073**	0.89	0.77; 1.01	0.0816
	OCGB^+^/IgG^+^/IgM^+^	1.44	1.22; 1.71	**< 1e-04**	0.95	0.81; 1.10	0.4876
TNFRSF10A	OCGB^+^/IgG^−^/IgM^−^	0.99	0.85; 1.15	0.8690	0.97	0.87; 1.09	0.6484
	OCGB^+^/IgG^+^/IgM^−^	1.35	1.17; 1.56	**<1e-04**	1.02	0.92; 1.14	0.6909
	OCGB^+^/IgG^+^/IgM^+^	1.43	1.22; 1.68	**<1e-04**	1.05	0.94; 1.19	0.3870
NfL	OCGB^+^/IgG^−^/IgM^−^	1.30	0.93; 1.84	0.1301	1.16	0.87; 1.56	0.3097
	OCGB^+^/IgG^+^/IgM^−^	1.79	1.30; 2.46	**0.0004**	1.47	1.12; 1.92	**0.00** **57**
	OCGB^+^/IgG^+^/IgM^+^	2.09	1.46; 2.98	**<1e-04**	1.69	1.25; 2.29	**0.0008**
GFAP	OCGB^+^/IgG^−^/IgM^−^	0.85	0.67; 1.08	0.1884	0.90	0.73; 1.11	0.3338
	OCGB^+^/IgG^+^/IgM^−^	1.18	0.95; 1.48	0.1313	1.08	0.89; 1.32	0.4260
	OCGB^+^/IgG^+^/IgM^+^	1.25	0.98; 1.59	0.0791	1.29	1.03; 1.61	**0.0** **253**
MOG	OCGB^+^/IgG^−^/IgM^−^	0.96	0.84; 1.10	0.5551	1.11	0.97; 1.27	0.1232
	OCGB^+^/IgG^+^/IgM^−^	1.07	0.95; 1.22	0.2568	1.30	1.15; 1.47	**<1e-04**
	OCGB^+^/IgG^+^/IgM^+^	1.05	0.92; 1.21	0.4749	1.37	1.19; 1.57	**<1e-04**

Abbreviations: Est. = estimate; Ig G/M/A_IF_ = immunoglobulin G/M/A intrathecal fraction; LP = lumbar puncture; OCGB = oligoclonal IgG band.

^+^: Presence of OCGB or IgG_IF_/IgM_IF_/IgA_IF_.

^−^: Absence of OCGB or IgG_IF_/IgM_IF_/IgA_IF_.

*Adjusted for age, sex, Qalb, and DMT category at LP.

**vs reference group OCGB^−^/IgG^−^/IgM^−^, respectively.

#### Serum

Consistent with the pattern observed in CSF, serum levels of NfL and GFAP were numerically increased in the presence of IgG^+^ and were maximal, as well as both significant with additional IgM^+^ in CSF (respective increase for NfL and GFAP were 1.7-fold and 1.3-fold). Similarly, MOG concentrations were increased 1.3-fold (*p* < 0.0001) in the OCGB^+^/IgG^+^/IgM^−^ category and 1.4-fold (*p* < 0.0001) in patients with MS with an additional intrathecal IgM synthesis (Table 2; Figure 2B).

### Time to Second Clinical Event in Patients With CIS

#### CSF

**Figure 3:**
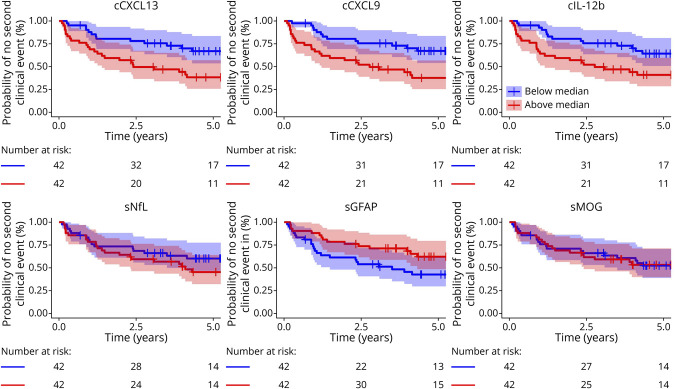
Time to Second Clinical Event in Patients With CIS In multivariable analyses adjusted for age, sex, Qalb, and DMTs, for each doubling of baseline CSF CXCL13 levels, the hazard ratio (HR) increased by 26% (HR 1.26; *p* = 0.007) and similarly for CXCL9 (HR 1.31; *p* = 0.013) and IL-12b (HR 1.43; *p* = 0.002). No such effects were found in serum, including NfL, GFAP, and MOG. CIS = clinically isolated syndrome; DMT = disease-modifying therapy; GFAP = glial fibrillary acidic protein; HR = hazard ratio; MOG = myelin oligodendrocyte glycoprotein; NfL = neurofilament light chain; Qalb = albumin quotient; s = serum.

In CSF, a shorter interval from the first to the second clinical event in patients with CIS was predicted by increased CXCL13, CXCL9, and IL-12b levels: for each doubling of baseline CXCL13 levels, the hazard ratio (HR) increased by 26% (HR 1.26; *p* = 0.0075), and similarly for CXCL9 (HR 1.31; *p* = 0.0134) and IL-12b (HR 1.43; *p* = 0.0017) (Figure 3; eTable 10).

#### Serum

Higher levels of VCAN (HR 0.34; *p* = 0.0158), CNTN2 (HR 0.49; *p* = 0.0083), and APLP1 (HR 0.39; *p* = 0.0186) were associated with a longer interval to the second clinical event (eTable 10). None of the other serum candidates, including NfL, GFAP, and MOG, predicted a shorter interval (Figure 3).

### EDSS Score at the First Demyelinating Event

In the CIS group (n = 110), CSF/blood samples were obtained a median of 18 days (IQR 9; 57) after the onset of symptoms from the first demyelinating event.

#### CSF

**Figure 4:**
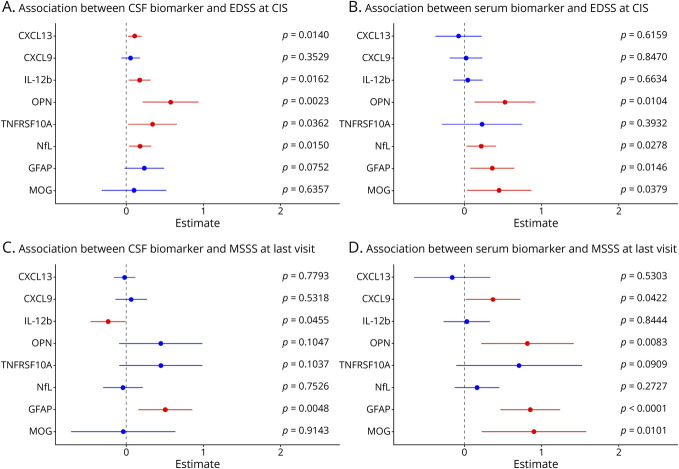
Multivariable Analyses Investigating the Associations of Biomarkers With EDSS Scores at CIS Onset in CSF (A) and Serum (B) and With MSSS at Last Visit in CSF (C) and Serum (D) (A) CSF: for each doubling of CXCL13 levels, the average EDSS score at CIS onset increased by 0.11 steps. Similarly, the following associations were observed: IL-12b: 0.18 steps. OPN: 0.58 steps; TNFRSF10A: 0.34 steps; NfL 0.18 steps. (B) Serum: a strong association with EDSS score at CIS onset was found for OPN, with a 0.52-step increase per doubling. NfL was also associated with higher EDSS values (0.22 steps), as was GFAP (0.36 steps) and MOG (0.45 steps). (C) CSF: higher GFAP levels prognosticated a higher future MSSS, per doubling of baseline levels the MSSS increased by 0.51 steps. (D) Serum: doubling of baseline serum CXCL9, OPN, GFAP, and MOG levels was associated with an increase of 0.37, 0.82, 0.85, and 0.90 steps in long-term disability scores. CIS = clinically isolated syndrome; EDSS = Expanded disability status scale score; GFAP = glial fibrillary acidic protein; MOG = myelin oligodendrocyte glycoprotein; MSSS = multiple sclerosis severity score; NfL = neurofilament light chain; OPN = osteopontin.

For each doubling of CXCL13 levels, the average EDSS score at lumbar puncture (LP) in patients with CIS increased by 0.11 steps (*p* = 0.014). Similarly, the following associations were observed: IL-12b: 0.18 steps (*p* = 0.0162); OPN: 0.58 steps (*p* = 0.0023); TNFRSF10A: 0.34 steps (*p* = 0.0362); NFL: 0.18 steps (*p* = 0.0150); COL4A1: 0.35 steps (*p* = 0.0201) (Figure 4A; eTable 11).

#### Serum

Similar to CSF, a strong association with EDSS scores at CIS onset was also found for OPN, with a 0.52-step increase per doubling (*p* = 0.0146). NfL was also associated with higher EDSS scores (est. 0.22, *p* = 0.0278), as was GFAP (est. 0.36, *p* = 0.012). Furthermore, doubling of MOG levels was associated with a 0.45-step increase in EDSS (*p* = 0.0379), again without corresponding changes in CSF (Figure 4B; eTable 11).

### Future MSSS

#### CSF

While higher IL-12b baseline levels were modestly associated with a lower future MSSS (est. −0.23; *p* = 0.045), increased GFAP predicted a higher future MSSS, per doubling of baseline CSF levels the MSSS increased by 0.51 steps (*p* = 0.0048). Furthermore, OPG was associated with a higher future MSSS (est. 0.63; *p* = 0.0337) (Figure 4C; eTable 12).

#### Serum

Doubling of baseline CXCL9 and OPN levels were associated with an increase of 0.37 (*p* = 0.0422) and 0.82 (*p* = 0.0082) MSSS steps, respectively. For GFAP, we observed an even more pronounced association in serum compared with CSF: per doubling of GFAP, there was an increase of 0.85 steps (*p* < 0.0001). Doubling of baseline MOG levels was also associated with an increase of 0.90 (*p* = 0.0101) in long-term disability scores (Figure 4D). In addition, VCAN (est. 1.06; *p* = 0.0154) and TNFSF13B (est. 0.93; *p* = 0.0177) were prominently associated with higher MS severity scores (eTable 12).

## Discussion

**Figure 5:**
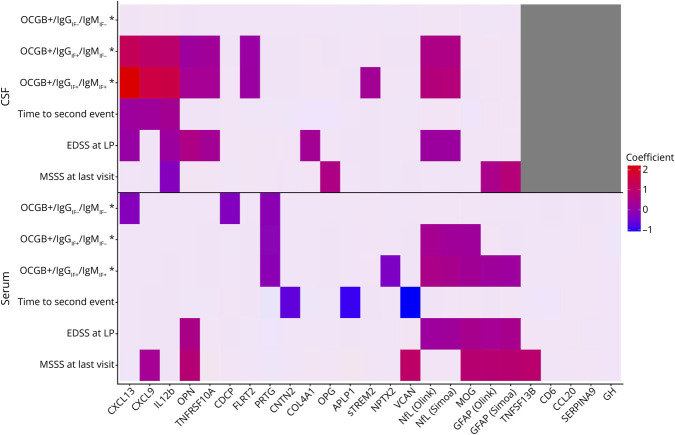
Summary Schematic of Associations Between Biomarkers and Various Outcomes in CSF and Serum in Patients With CIS/MS Significant associations are color-coded according to the magnitude of the coefficient. *vs OCGB^−^/IgG_IF_^−^/IgM_IF_^−^. Analytes are described in eTable 1; CIS = clinically isolated syndrome; EDSS = Expanded Disability Status Scale; LP = lumbar puncture; MSSS = multiple sclerosis severity score.

We identified 8 biomarkers, CXCL13, CXCL9, IL-12b, OPN, TNFRSF10A, NfL, GFAP, and MOG, in CSF and/or serum from a preselected panel of 23 candidates,^6,7^ based on (a) analytical reproducibility and molecular stability, (b) elevated levels in CIS and MS, and (c) consistent associations with clinical outcome measures and their role for B-cell and T-cell recruitment and activation in the CNS during MS.^6,7,16-18^ To facilitate an integrated interpretation of our findings, we present a summary schematic illustrating the associations between biomarkers and various outcomes in CSF and serum (Figure 5).

The increase of cytokines CXCL13, CXCL9, and IL-12b exhibited in CSF across most CIS/MS groups was strongly intercorrelated. Their quantitative association, in function of the intrathecal IgG synthesis pattern, with peak levels in patients with intrathecal IgM synthesis, an established marker of higher MS disease activity^15,19,20^ establishes them as part of a proinflammatory network in MS pathogenesis. Recent work further revealed strong, consistent correlations between these cytokines and complement system components and activation products in MS, underscoring their pathophysiologic significance.^21,22^

Accordingly, elevated baseline CSF levels of these cytokines were associated with a shorter time to second clinical event, in line with previous studies demonstrating that elevated CSF CXCL13 levels reflect present and predict future disease activity and conversion from CIS to MS.^17,23,24^ CSF IL-12b and CXCL9 levels have consistently been reported as being prominently increased in patients with MS.^16,25^ It is important to note that we observed extensive changes for cytokines in CSF but no elevations or meaningful associations in serum, highlighting once again that inflammation in MS is compartmentalized within the CNS.

NfL is a well-established biomarker indicative of neuroaxonal damage, GFAP reflects reactive astrogliosis and/or astrocytic injury, and MOG is a CNS-specific protein of the myelin sheath.^4,26,27^ The Olink platform, as measured using the MSDA Test custom assay panel, is now the first to quantitate the release of MOG into serum, allowing comprehensive monitoring of all 3 main CNS cell types, neurons, astrocytes, and oligodendrocytes, through minimally invasive sampling.

NfL has gained recognition in recent years as a real-time marker of disease activity in MS.^4,28,29^ Moreover, serum NfL serves as a marker of therapeutic response, with concentrations shown to decrease under disease-modifying therapies.^3^ Consequently, NfL is increasingly used as an end point in clinical trials and for monitoring treatment response in individual patient care.^30,31^ Complementary to NfL, GFAP has recently emerged as a second key biomarker in MS, predominantly associated with disease severity and future progression.^5,32,33^

Current results are fully congruent with these findings, and for NfL, expand this association to CSF.

Although there is a good correlation of NfL and GFAP levels between serum or plasma and CSF in our results and in earlier studies,^4^ the observation that higher serum, but not CSF, levels of GFAP were associated with an intrathecal IgM synthesis, as well as higher EDSS scores in patients with CIS, is counterintuitive. This finding was reproducible across different assay platforms and hence may not be due to technical-analytical issues of measurement. However, GFAP levels in CSF were associated with higher MS severity scores, although the effect size was less pronounced than for serum levels. There is a need to elucidate this discrepancy, as we hypothesize that the composition of isomers and degradation products of GFAP and their clearance differs between the 2 fluid compartments, eventually leading to an incongruent signal pattern. A similar phenomenon has been described for GFAP in Alzheimer disease. Consistent with our observations, it was previously reported that differences in blood GFAP levels between patient groups were more pronounced than those in CSF, and the associations between plasma GFAP and amyloid-β biomarkers reflecting amyloid pathology were stronger than those observed in CSF. These findings should be interpreted with caution and will require further confirmation in independent cohorts directly comparing the 2 matrices.^34^

MOG levels are believed to reflect CNS-compartmentalized demyelinating damage.^26^ The availability of an assay to quantitate MOG now opens the possibility of a 'liquid biopsy' to elucidate this pathology. However, we observed increased serum, but not CSF, levels of MOG and levels of both compartments were not correlated. Similar to the discrepancy observed between CSF and blood compartment for GFAP levels, serum MOG levels were elevated in patients exhibiting an intrathecal IgM synthesis and showed strong associations with EDSS scores at lumbar puncture in the CIS subgroup, as well as with future long-term disability measured by the MSSS, while this was not the case for CSF levels. These associations were comparable in magnitude to those observed for serum GFAP. Given the distinct pathophysiologic background, MOG may represent a valuable complementary marker in the future. However, at this stage, MOG does not provide significant additional clinical information beyond GFAP; this remains preliminary and requires further validation.

OPN is a cytokine implicated in cellular immune responses.^35^ We found modestly elevated OPN levels in the CSF of patients with RRMS and PPMS and a modest association with an intrathecal IgM synthesis, but none for the risk of future relapses as reported earlier.^35,36^ The correlation of elevated OPN concentrations in both CSF and serum with higher baseline EDSS scores in patients with CIS and the increase of serum OPN levels predicting long-term MS disability is novel, a finding not previously reported.^25,35,36^ Further investigation is needed whether OPN provides additional information about disease severity, but in synopsis, the current potential appears relatively weak.

TNFRSF10A is involved in mediating apoptosis, and in our study, CSF levels of TNFRSF10A were significantly elevated in patients with PPMS, while consistent upward trends were observed in CIS, RRMS, and SPMS subgroups. In addition, higher CSF TNFRSF10A levels correlated with increased EDSS scores in patients with CIS but showed no association with future MSSS. From a pathophysiologic standpoint, these findings provide novel insights that have not been previously reported^6,37^ but do not reveal a clear concept of the function of TNFRSF10A in MS.

For the other biomarkers analyzed, our findings suggest a more limited perspective for their clinical utility in MS. Previous studies have shown that VCAN does not exhibit significant associations with radiographic or clinical indicators of disease activity, such as gadolinium-enhancing lesions or relapse rates.^6,7^ Similarly, our study did not reveal consistent or clinically meaningful results for VCAN compared with other candidates, although a notably strong association with future MSSS was observed. GH and COL4A1 were previously excluded because of issues with assay precision and diurnal variability during analytical validation.^7^ In our study, CSF levels of BAFF, SERPINA9, CCL20, CD6, and GH frequently fell below the lower limit of quantification (LLOQ) on the multiplex assay, preventing reliable analysis of these candidates in CSF. Furthermore, no compelling results were found in serum for these markers, nor for CNTN2, OPG (TNFRSF11B), CDCP1, APLP1, FLRT2, and PRTG. Contrary to our findings, Jalaleddini et al.^38^ identified PRTG, alongside serum GFAP, as predictor of future disability progression. In addition, sTREM2, a potential marker of microglial activation, did not demonstrate convincing results in our cohort.

Limitations of our study include the relatively small number of patients with primary progressive MS, and the fact that, owing to the analytical methods used, several analytes in CSF were below the LLOQ. Furthermore, the antibody-based measuring technique is blind to the quantitative distribution of isoforms and degradation products of respective markers in respective biofluid compartments, which is a prerequisite for an in-depth understanding of their pathophysiologic function and interpretability of measurements. Although the MSDA panel was originally discovered and validated in serum,^6,7^ we ensured the technical reliability of our measurements by performing characterization experiments to evaluate assay performance in CSF prior to study initiation. While the biological relevance of these proteins remains compelling within the CNS, this work does not constitute a validation of the MSDA composite algorithm in CSF and should not be interpreted as establishing clear clinical equivalence between the 2 matrices. Furthermore, ethnicity and socioeconomic status were not systematically assessed in this study and should be considered a limitation because these factors may influence biomarker distributions and disability outcomes.

In conclusion, the cytokines CXCL13, CXCL9, and IL-12b in CSF consistently stand out as robust predictors of disease activity, particularly with respect to focal inflammation. They show strong intercorrelations and are closely associated with intrathecal IgM synthesis, which is itself a powerful predictor of MS disease activity. This relationship is consistent with their established pathophysiologic role in orchestrating and regulating the cellular immune response driving focal inflammation. Because the assessment of intrathecal IgM synthesis remains difficult to standardize, these cytokines, individually or in combination, may represent more quantifiable and practical biomarkers for predicting disease activity.

NfL in both CSF and serum is already recognized as a reliable marker of neuroaxonal damage and serves as a valuable tool for therapy monitoring. Serum MOG and GFAP, which indicate both disease severity and myelin and astrocytic damage, respectively, with GFAP being much further advanced, may serve as valuable additions for clinical practice.

## Glossary

APLP1: amyloid-beta precursor-like protein 1
BAFF (TNFSF13B): B-cell activating factor (tumor necrosis factor superfamily member 13B)
CCL20: CC motif chemokine ligand 20
CD6: cluster of differentiation 6
CDCP1: CUB domain-containing protein 1
CIS: clinically isolated syndrome
COL4A1: collagen type IV alpha 1 chain
CNTN2: contactin 2
CXCL9: CXC motif chemokine ligand 9
CXCL13: CXC motif chemokine ligand 13
CV: coefficient of variation
DMT: disease-modifying therapy
EDSS: Expanded Disability Status Scale
FLRT2: fibronectin leucine-rich transmembrane protein 2
GFAP: glial fibrillary acidic protein
GH: growth hormone
HR: hazard ratio
IF: intrathecal fraction
Ig: immunoglobulin
IL-12b: interleukin-12 subunit beta
INDCs: inflammatory neurologic disease controls
IQR: interquartile range
LLOQ: lower limit of quantification
LP: lumbar puncture
MOG: myelin oligodendrocyte glycoprotein
MS: multiple sclerosis
MSDA: multiple sclerosis disease activity (Test)
MSSS: Multiple Sclerosis Severity Score
NfL: neurofilament light chain
NPTX2: neuronal pentraxin 2
OCGBs: oligoclonal IgG bands
OPG (TNFRSF11B): osteoprotegerin
OPN: osteopontin
PEA: proximity extension assay
PIRA: progression independent of relapse activity
PPMS: primary progressive multiple sclerosis
PRTG: protogenin
QC: quality control
Qalb: albumin quotient
RRMS: relapsing-remitting multiple sclerosis
SCs: symptomatic controls
SERPINA9: serpin family A member 9
SMSC: Swiss multiple sclerosis cohort
SPMS: secondary progressive multiple sclerosis
sTREM2: soluble triggering receptor expressed on myeloid cells 2
TNFRSF10A: tumor necrosis factor receptor superfamily member 10A
TNFSF13B: tumor necrosis factor superfamily member 13B (BAFF)
ULOQ: upper limit of quantification
VCAN: versican
